# Why another conference on personalized medicine?

**DOI:** 10.3325/cmj.2012.53.205

**Published:** 2012-06

**Authors:** Soulla Louca, Roland Pochet, Dieter Schinzer

**Affiliations:** COST DC Information and Communications Technologies; COST DC Biomedicine and Molecular Biosciences; COST DC Chemistry and Molecular Sciences and Technology

Personalized medicine, a discipline enabled by genetic, biopharma, diagnostic, and information and communications (ICT) technologies attempts to tailor disease treatment to individual patients. Personalized medicine is rapidly emerging as a state-of-the-art approach to diagnostics and therapeutics and is beginning to revolutionize our health care system, promising better medicine for all.

COST – European Cooperation in Science and Technology – opened up the field of personalized medicine in 1986 when, as part of the Biomedicine and Molecular Biosciences Domain, it sanctioned a research network (aka COST Action) to recruit trial subjects based on differences in metabolic capacity (COST Action B1, 1986). Since then, progress in technology, along with a growing understanding of individual susceptibility to disease and treatment effects, has led to new concepts in translational science such as the Virtual Patient Model. These developments are transforming the way we think about health and disease.

Interdisciplinary methods are facilitating the creation of integrated data sets that incorporate biological, chemical, and clinical observations through the use of sophisticated software and hardware. Mathematics, physics, and chemistry are all employed in all aspects of ICT, including gathering data, simulating biological processes, and visualizing the system as a whole.

Despite the advances, considerable challenges remain in realizing the full potential of personalized medicine, including creating a greater awareness among global stakeholders. Initiatives in Europe, including the UK’s Stratified Medicine Innovation Platform, Sweden’s Biobank Program, BIOMEDREG in the Czech Republic, and the Munich Biotech Cluster in Germany, are working toward this goal, but the efforts are still modest. Our conference aims to highlight the current activities, along with future possibilities, to a broader audience.

Overcoming identified obstacles to making personalized medicine more available for patients requires a focus on basic, translational, and regulatory science, especially in the areas of ethical, legal, and social issues. Improvement in patient care must remain a priority throughout the collection and use of information on an individual patient’s genome and its downstream products including transcriptomes, proteomes, and metabolomes. This will require a significant exploration of strategic relationships between all interested parties with proposals for further interdisciplinary collaborations involving mathematics, physics, chemistry, and ICT.

Connecting high-quality trans-disciplinary scientists through programs such as COST can support capacity building and increase the impact of personalized medicine research on regulatory bodies, decision makers, pharmaceutical companies, and payers. Such collaborations could enable breakthrough scientific developments, lead to new concepts and products, and contribute to Europe’s strength in research and innovation, while reforming the health care system.

With this vision in mind, the Chairs of the COST Domains of ICT, Biomedicine and Chemistry used their privileged positions to gather their corresponding scientific communities together with science policy makers (António Correia de Campos MEP – European Parliament, Irene Norstedt – European Commission, Stavros Malas – Minister of Health of Cyprus) and patients’ associations (Mary Baker – former President of European Parkinson Disease Association) for the upcoming conference. We believe the three domains have succeeded in putting together the necessary stakeholders to prepare the ground for significant advancement in science and technology, and for critical influence on science policy.

The success in attracting such a significant and diverse cadre of policy makers, patients, and top scientists is the full justification for another conference on personalized medicine. The conference will take place from June 17 to 22, 2012 at Golden Bay Beach Hotel in Larnaca, Cyprus (*http://www.cost.eu/events/pemed*).

This event will be not only published in proceedings but will also be featured on YouTube.

